# Loss of Oxygen Atoms on Well-Oxidized Cobalt by Heterogeneous Surface Recombination

**DOI:** 10.3390/ma16175806

**Published:** 2023-08-24

**Authors:** Domen Paul, Miran Mozetič, Rok Zaplotnik, Jernej Ekar, Alenka Vesel, Gregor Primc, Denis Đonlagić

**Affiliations:** 1 Jozef Stefan Institute, Jamova Cesta 39, 1000 Ljubljana, Slovenia; 2 Jozef Stefan International Postgraduate School, Jamova Cesta 39, 1000 Ljubljana, Slovenia; 3 Faculty of Electrical Engineering and Computer Science, University of Maribor, Koroska Cesta 46, 2000 Maribor, Slovenia

**Keywords:** heterogeneous surface recombination, recombination coefficient, cobalt, cobalt oxide, temperature dependence, pressure dependence, plasma, oxygen

## Abstract

Calorimetry is a commonly used method in plasma characterization, but the accuracy of the method is tied to the accuracy of the recombination coefficient, which in turn depends on a number of surface effects. Surface effects also govern the kinetics in advanced methods such as atomic layer oxidation of inorganic materials and functionalization of organic materials. The flux of the reactive oxygen atoms for the controlled oxidation of such materials depends on the recombination coefficient of materials placed into the reaction chamber, which in turn depends on the surface morphology, temperature, and pressure in the processing chamber. The recombination coefficient of a well-oxidized cobalt surface was studied systematically in a range of temperatures from 300 to 800 K and pressures from 40 to 200 Pa. The coefficient increased monotonously with decreasing pressure and increasing temperature. The lowest value was about 0.05, and the highest was about 0.30. These values were measured for cobalt foils previously oxidized with oxygen plasma at the temperature of 1300 K. The oxidation caused a rich morphology with an average roughness as deduced from atomic force images of 0.9 µm. The results were compared with literature data, and the discrepancy between results reported by different authors was explained by taking into account the peculiarities of their experimental conditions.

## 1. Introduction

Precise oxidation of various materials has attracted the attention of numerous authors involved across multiple research fields, from atomic layer oxidation [1,2] to polymer activation [3,4,5], sterilization [6,7], and degreasing of inorganic materials [8,9]. Oxygen under ambient conditions is composed of two-atom molecules, which are not very chemically reactive at room temperature [10,11]. Faster and more controlled oxidation is obtained by reactive oxygen, such as that of ozone, superoxide, and neutral oxygen atoms in the ground state [12,13]. All of these reactive oxygen species interact with solid materials at various rates, depending on the material type and temperature [14]. While both ozone and superoxide are challenging to produce and sustain at large concentrations, neutral oxygen atoms in the ground state are rather easily produced by electron-impact dissociation upon plasma conditions, because the dissociation energy of an oxygen molecule is only 5.2 eV [15].

The recombination in the gas phase cannot occur via collision between two atoms due to the conservation of energy and momentum. The conservation of energy and momentum can be satisfied only by three-body collisions. The probability of three-body collisions increases as a square of the density of atoms in the gas phase. It is as large as about 1 MHz at atmospheric pressure and only about 1 Hz at 1 mbar [16]. Neutral oxygen atoms are very stable at low pressures as long as the loss is due to the gas phase reactions. The major mechanism of association between atoms and stable molecules at low pressure is heterogeneous surface recombination. This reaction requires at least a moderate lifetime of atoms adsorbed on the material surface. Different materials provide various lifetimes for O atoms, depending on the chemical interaction between oxygen atoms and the substrate.

Inert materials will exhibit short lifetimes and, thus, a low recombination coefficient. On the other hand, reactive materials, which tend to form oxides of moderate binding energy, will exhibit large recombination coefficients. The recombination coefficient depends not only on the type of material but also on its roughness, temperature, or pressure [17]. Of particular interest are materials whose recombination coefficient does not depend so much on the temperature or pressure in the range, which is useful for the processing of materials by oxygen atoms, for example, atomic layer oxidation of metals [18] and functionalization of polymers with polar functional groups [19].

Transition metal oxides are candidates for materials with high recombination coefficients because they form various oxides whose binding energy is just a small amount of eV [20]. These have attracted the attention of numerous authors, who have reported various values. A material of particular interest is cobalt because it forms only one type of oxide, which is stable in a range of temperatures up to about 900 K [20]. The stable form of cobalt oxide is CoO, often referred to as Co (II) oxide. This oxide transforms to $Co_{3}O_{4}$ at elevated temperatures but decomposes back to Co (II) oxide at about 1200 K [21].

Additionally, cobalt is a highly sought-after catalytic material in various industrial applications [22]. Along with applications in renewable energy conversion, and oil purification, cobalt is a low-cost alternative to other catalytic materials, such as precious metals. In all of the above-mentioned processes, the presence of oxygen, especially the more reactive neutral oxygen atoms can alter the surface of the catalyst.

Probably the first report about the recombination of neutral oxygen atoms on the surface of cobalt oxide was published in 1959 in a classical paper by Greaves and Linnett [23]. The authors used the Wrede–Harteck gauge to estimate the recombination coefficient. The reported value was approximately $5\times 10^{-3}$. This value was measured at room temperature and at an oxygen pressure of 650 Pa.

A few years later, Dickens and Sutcliffe measured the recombination coefficients of selected metal oxides versus material temperatures [24]. At room temperature, the recombination coefficient for $Co_{3}O_{4}$ was approximately $2.5\times 10^{-3}$. The authors also reported a rise in the conductivity of cobalt oxide due to exposure to oxygen atoms. They performed measurements at a pressure as high as 13,333 Pa. They found an exponential increase in the recombination coefficient versus the temperature for many transition metal oxides, but not for $Co_{3}O_{4}$. Nevertheless, the recombination coefficient increased monotonously with increasing temperature and then reached a value of approximately 0.25 at the maximum temperature probed by [24], i.e., 625 K.

Melin and Madix [25] performed experiments in a sophisticated version of the reactor used originally by Greaves and Linnett [23]. Surprisingly enough, they reported an order of magnitude larger coefficient, i.e., $7.5\times 10^{-2}$, at room temperature. They did not report the type of oxide formed on the metallic surface upon exposure to neutral oxygen atoms. More recently, Guyon et al. [26] used actinometry to estimate the recombination coefficients of selected metal oxides. They reported the coefficient for CoO of $2.9\times 10^{-2}$ at 300 K and $3.4\times 10^{-2}$ at 473 K. Unlike Dickens and Sutcliffe [24], they found a temperature (T) dependence of the recombination coefficient (γ) to be:

(1) $$\gamma =\frac{T_{0}}{T}e^{-\frac{E_{a}}{RT}}\;,$$

where $T_{0}$ was a characteristic temperature, $E_{a}$ was the activation energy of the surface reaction, and R was the gas constant. However, they performed measurements at only three different temperatures (i.e., 300, 385, and 473 K). Cvelbar et al. [27] compared several catalytic materials by exposing them to the oxygen with a dissociation fraction of the order of 10% in a broad range of pressures between 5 and 400 Pa. While the coefficient for copper was found to be independent of pressure, complex behavior was observed for cobalt. Namely, a maximum coefficient of $14\times 10^{-2}$ was observed at around 30 Pa, and the coefficient decreased almost exponentially at higher pressures and stabilized at the value of about $8.5\times 10^{-2}$ in the range of pressure between 200 and 400 Pa. At pressures lower than 30 Pa, however, the recombination coefficient decreased with decreasing pressure.

This brief literature survey indicates the inadequate knowledge of the recombination probability for oxygen atoms on the CoO surface. The discrepancy between the reported results may be due to several reasons, such as the different experimental methodologies used by authors and the different surface conditions of the material, i.e., the different morphology of samples. It is known that the measured recombination coefficient increases with increasing roughness [28,29], mainly because of the larger available surface area as compared with the geometrical area. The largest recombination coefficient reported in reliable literature is that of carbon nanowalls [30], with the very large coefficient explained by trapping oxygen atoms into the gaps between neighboring walls, thus causing numerous collisions of atoms on the material surface.

We performed experiments using samples of equal morphology and structure to clarify the temperature and pressure dependencies of the recombination coefficient for oxygen atoms on the surface of well-oxidized cobalt. A stable oxide film was prepared by exposing the high-purity cobalt disk to a mixture of molecular and atomic oxygen at a large atom flux and temperatures as high as 800 K. According to the knowledge of the stability of cobalt oxides [21], the surface composition should be independent of the flux of reactive oxygen species in a temperature range from room temperature to about 900 K. The purpose of this study was to determine the interaction of neutral oxygen atoms with oxidized cobalt under experimental conditions commonly used in plasma laboratories around the world, as both oxygen plasma and cobalt are often used without sufficient understanding of their interactions.

## 2. Experimental

**Figure 1:**
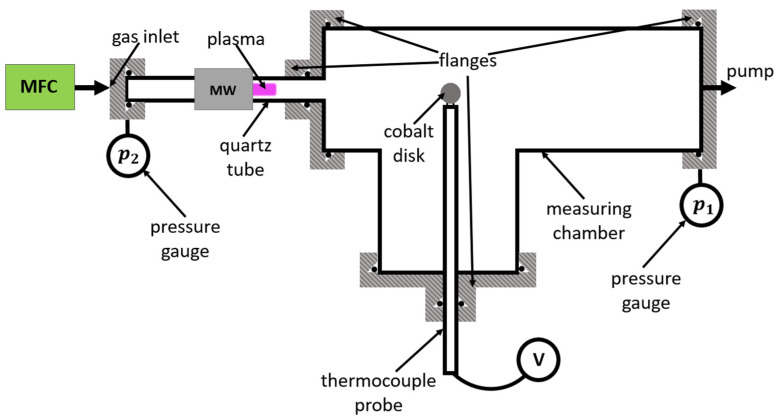
Schematic of the experimental setup that enabled exposure of a cobalt disk to a constant flux of O_2_ and O.

The pressure and temperature evolution of the recombination coefficient for cobalt oxide was measured in the experimental setup shown schematically in Figure 1. First, molecular oxygen, supplied via the mass flow controller (MFC), entered a quartz tube, where it was partially dissociated in gaseous plasma, resulting in a mixture of O_2_ and O. The plasma was sustained by a microwave discharge operating in the surfatron mode [31]. Such sources of neutral oxygen atoms are widely used in laboratories worldwide because of their robustness, stability, and ease of operation. The exact kinetics of plasma radicals was later reported by several authors, including Kutasi et al. [31], Ricard et al. [32], and Guerra et al. [33].

The source of oxygen atoms was connected to a measuring chamber, where a cobalt disk was positioned. There was a drift of gas from the oxygen flask through the discharge tube and the measuring chamber due to the continuous pumping of the experimental system. Our system was pumped with an Edwards E2M80 two-stage rotary pump with a nominal pumping speed of 80 $m^{3}/h$ and ultimate pressure below 1 Pa. Molecular oxygen was introduced into the experimental system through a calibrated flow controller. As there was no detectable leakage of the vacuum system, the mass flow remained constant through all the vacuum elements between the flow meter and the pump, but the volume flow increased towards the pump. A pressure gradient was established along the narrowest vacuum elements. Because all components except the discharge tube have an inner diameter of 3.6 cm or more, practically the entire pressure gradient was established in the discharge tube. The discharge tube had an inner diameter of 6 mm and a length of 41.5 cm. Two absolute vacuum gauges were mounted on either side of the discharge tube, as shown in Figure 1. The discharge was powered with a microwave generator (Sairem GMS200WSM, Décines-Charpieu, France) with a maximum output power of 200 W. The reflected power was also measured, so all results presented in this paper are versus the difference between forward and reflected power.

The oxygen atom density in the measuring chamber was determined by the Šorli–Ročak method [34]. Briefly, this is a reliable method for the determination of the absolute density of neutral atoms in low-pressure systems. The accuracy of the method is about 20%. Details regarding this method are disclosed in our recent paper [35].

**Figure 2:**
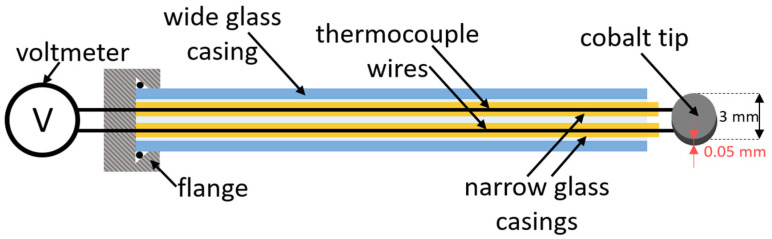
Schematic of the disk installation.

High-purity (99.99%) cobalt foil of a thickness of 0.05 mm was purchased from Goodfellow (Huntingdon, UK). Small disks of a diameter of 3 mm were cut from the foil and spot-welded to chromel–alumel thermocouple wires of a diameter of 0.25 mm. Each wire was placed into a very narrow glass tube to prevent extensive loss of O atoms on the wires themselves. Wires were connected to a voltmeter through a vacuum-tight feedthrough. The dimensions of the cobalt disk and the supporting wires and tubes are shown in Figure 2.

The disk shown in Figure 2 was thoroughly oxidized before measuring the recombination coefficient. The oxidation was performed by placing the disc into a rather dense oxygen plasma sustained by inductively coupled radiofrequency discharge in the H-mode. Details about this type of plasma are described elsewhere [36]. The disk heated up to about 1300 K in radiofrequency plasma. The disk was kept in plasma for about 10 min to form a stable oxide film. All experiments for measuring the recombination coefficients were performed at much smaller temperatures, i.e., up to 800 K, so it is believed that the surface structure, composition, and morphology did not change during these measurements.

## 3. Results and Discussion

### 3.1. Characterisation of the Discharge System

**Figure 3:**
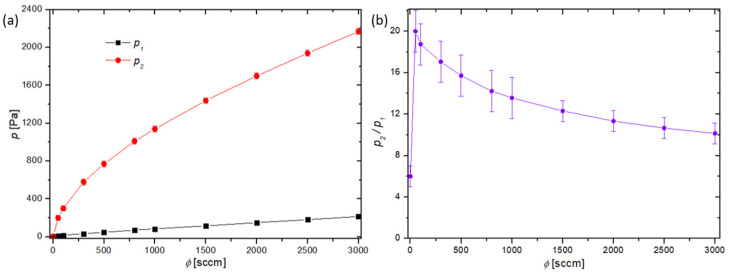
(**a**) The oxygen pressure on the high-pressure side ($p_{2}$) and low-pressure side ($p_{1}$) vs. the gas flow. (**b**) The ratio of pressures from each side of the discharge tube ($p_{2}/p_{1}$) vs. the gas flow.

Determination of the loss of oxygen atoms on the cobalt disk requires detailed characterization of oxygen flow rates and fluxes of oxygen atoms to the cobalt surface as well as any gradients in the system. After evacuating the system down to the ultimate pressure, oxygen was leaked through the mass flow controller during continuous pumping, establishing pressure gradient along the discharge tube. The pressures at both sides of the discharge tube were measured versus the gas flow and the results are shown in Figure 3a. Figure 3b shows the ratio of pressures at each end of the discharge tube versus the gas flow. One can observe that the pressure ratio slowly decreases with increasing flow rate. This observation is explained by the way in which the conductivity of the tube increases with increasing gas pressure.

This pressure gradient caused a drift of gas through the discharge tube. The gas velocity could be estimated, taking into account the results of Figure 3, and the gas flow rate determined by the flow controller, using the standard formula where the gas flow (in standard cubic centimeters (sccm)) is a constant at any given point in the system:

(2) $$v_{drift}=\frac{\phi_{sccm}10^{-2}}{1.425\cdot 6\;p\;S_{quartz}}\;,$$

where $\phi_{sccm}$ is the gas flow rate (in sccm units), p is the pressure at a given position in the system, and $S_{quartz}$ is the discharge tube cross-section. While the drift velocity is moderate at the entrance to the discharge tube, it is over 100 m/s at the exhaust of the measuring chamber. The sound velocity in gas limits the maximum drift velocity. Such a rapid transport of gas is essential to ensure a rather large density of atoms in the measuring chamber because the gas residence time in the discharge tube (marked as “quartz tube” in Figure 1) depends on the gas velocity. If the drift velocity were approaching zero, the transport of atoms from the plasma in the discharge tube to the position of the cobalt catalyst would have been by diffusion, so the atoms would have been lost during their collisions with the quartz tube surface.

**Figure 4:**
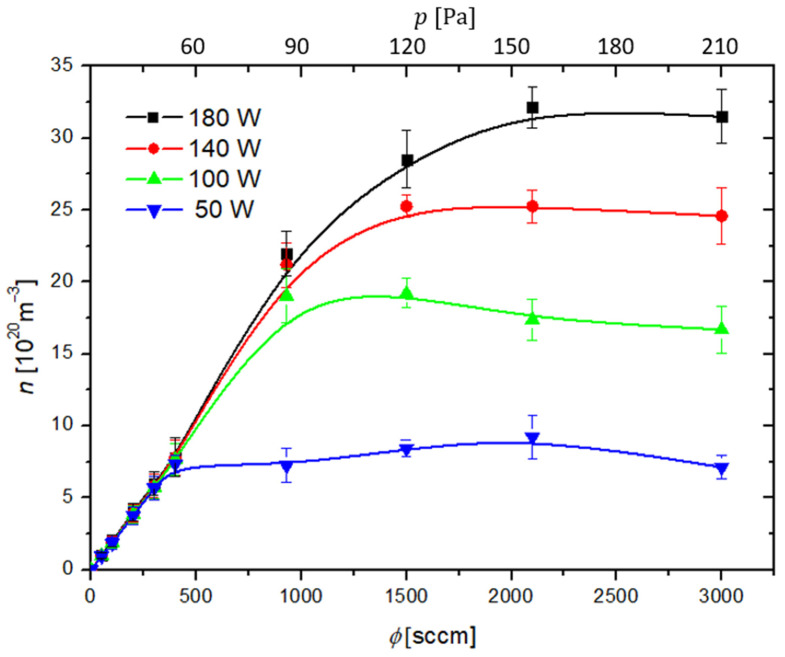
Oxygen atom density is measured at the position of the cobalt catalyst versus gas flow rate (ϕ) and pressure (p) inside the system at different discharge powers.

The density of oxygen atoms in the measuring chamber at the position of the cobalt catalyst was measured using the Šorli–Ročak method [34] and is plotted versus the oxygen flow and pressure in Figure 4. There are several curves corresponding to different discharge powers. As expected, the lowest oxygen atom density is at the lowest discharge power of 50 W. The oxygen atom density does not depend much on the discharge power at low flow rates, but it increases with increasing power at elevated flow rates, i.e., above 500 sccm. This is explained by the fact that both the length of the plasma column in the discharge tube and the density and/or temperature of the electrons increase with increasing discharge power [31,37,38]. It is important to stress that the densities provided in Figure 4 are at the position of the catalytic disk (Figure 1). As there is a pressure gradient along the discharge tube (Figure 3a), and the dissociation fraction inside plasma is at least as large as in the measuring chamber, the oxygen atom density in plasma is several times larger than in the measuring chamber. It is reasonable to assume a constant gradient of gas pressure inside the discharge tube, so the actual pressure at the position where glowing plasma is sustained can be estimated from Figure 3.

### 3.2. Determination of the Loss of Atoms on a Catalyst Material

Once the O-atom density in the measuring chamber is known, it is possible to determine the loss of atoms on a surface of any material mounted at the position marked as “cobalt disk” in Figure 1. A cobalt disk was connected to thermocouple wires as shown in Figure 2 and heated up to the temperature of about 1300 K in radiofrequency plasma for about 10 min to form a stable oxide film, as explained above. Such a well-oxidized disk was then mounted in the experimental system shown in Figure 1.

**Figure 5:**
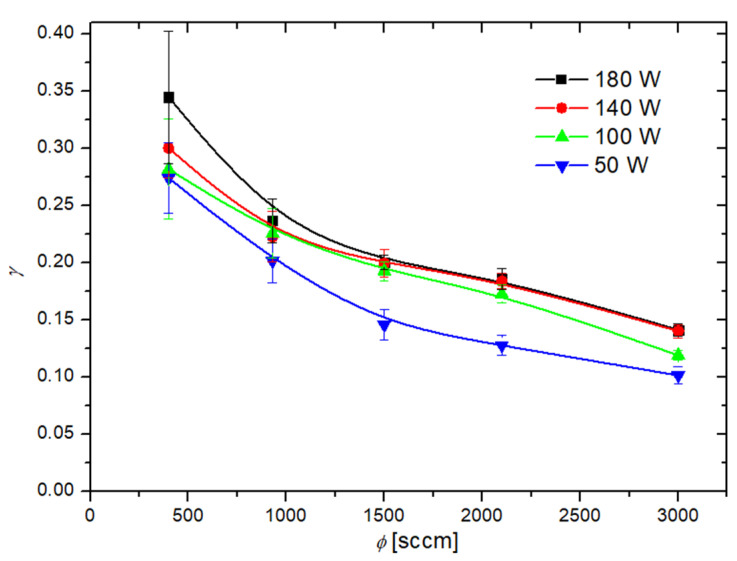
Recombination coefficient (γ) versus gas flow rate (ϕ) at various discharge powers. The error bars represent the statistical error only, as the measurements were repeated several times at identical conditions.

The recombination coefficient of the oxidized cobalt was determined as follows: the oxidized cobalt disk was at room temperature (about 300 K), before igniting the discharge. Despite the adiabatic expansion due to the pressure gradients (Figure 3), the gas temperature at the position of the cobalt disk was not much different from the ambient temperature. Once the discharge was ignited, the catalyst started heating due to the heterogeneous surface recombination of oxygen atoms into oxygen molecules. This reaction is highly exothermic, because the potential energy of an oxygen atom in the ground state is 2.6 eV, while the potential energy of O_2_ molecules in the ground electronic state and room temperature is practically 0 [39]. Because the supply of oxygen atoms into the measuring chamber is constant at a given flow rate and discharge power, the power dissipated on the catalyst surface due to the surface association is also constant. Within several seconds, the cobalt disk assumes a constant temperature, where the heating by heterogeneous surface recombination is equal to the cooling by any process, including the processes of grey body radiation, the thermal conduction of the thermocouple wires and the surrounding gas, and cooling by gas drift. The constant temperature after prolonged exposure of the disc to O atoms depends on the flux of oxygen atoms on the surface and the recombination coefficient. When the discharge is turned off, the disc temperature starts decreasing because of the absence of heating. The recombination coefficient (γ) is calculated from the measured absolute value of the time-derivative of the temperature of the disk (dT/dt) as:

(3) $$\gamma =\frac{8\;m\;c_{p}}{v\;W_{D}\;A\;n}\frac{dT}{dt}\;,$$

where m is the mass of the cobalt disk, $c_{p}$ is the specific heat of cobalt, v is the average value of the gas random velocity, $W_{D}$ is the dissociation energy of an oxygen molecule, A is the geometric surface area of the cobalt disk, and n is the density of neutral oxygen atoms at the position of the cobalt disk. The recombination coefficient versus the oxygen flow rate is shown in Figure 5. The discharge power is the parameter. Figure 5 indicates some dependencies, particularly the pressure dependence of the recombination coefficient (Figure 3), but according to previous authors, the coefficient should also depend on the temperature of the cobalt catalyst. Furthermore, the equilibrium temperature of the cobalt disc upon heating by the surface recombination of O atoms depends on the pressure, so Figure 5 is not valid for the determination of the pressure and temperature dependencies.

**Figure 6:**
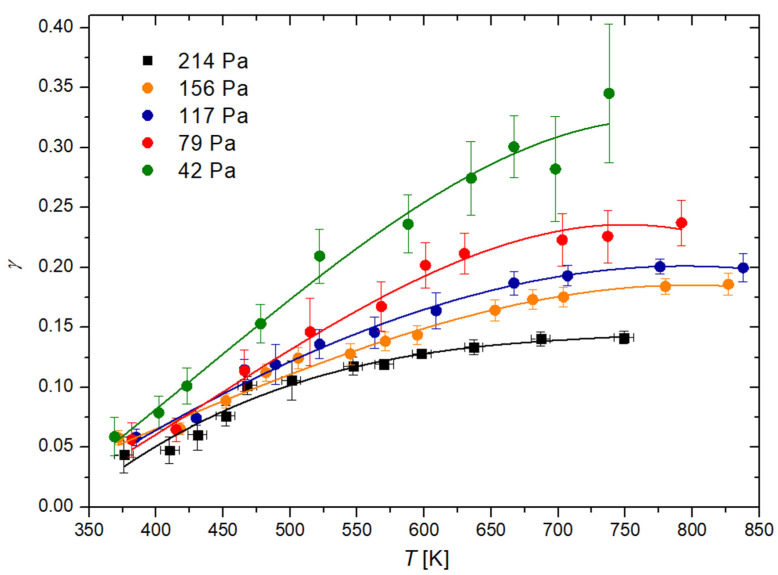
Recombination coefficient (γ) of the cobalt disk with respect to the temperature (T) of the cobalt disk at various pressures inside the system.

**Figure 7:**
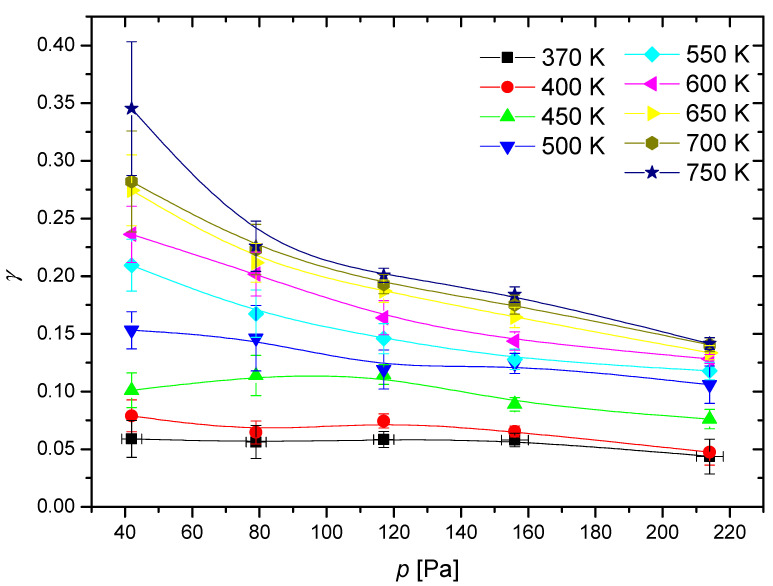
Recombination coefficient (γ) of the cobalt disk versus pressure (p) inside the system at various temperatures of the cobalt disk.

To determine the temperature dependence, the recombination coefficient is plotted in Figure 6 versus the maximum temperature of the catalytic disk, where the discharge pressure in the measuring chamber is the parameter. The results of Figure 5 were also used to determine the variation of the recombination coefficient versus the pressure in the measuring chamber. Figure 7 shows the values with the catalyst temperatures as the parameter. Figure 6 indicates a monotonous increase of the recombination coefficient with the increasing temperature of the cobalt disc. This observation is found with Dickens et al. [24] as well as Guyon et al. [26]. As mentioned earlier, Guyon et al., found a logarithmic increase in the recombination coefficient, but their results are based on only three measured points. In contrast, Dickens et al. found a complex behavior; in particular, a rather large increase of the recombination coefficient with increasing temperature from room temperature up to 500 K, but a marginal increase at temperatures above 500 K. By considering the results of Figure 6, the observations of Dickens et al., are closer to the measured temperature dependence of the recombination coefficient, as Figure 6 indicates stabilization of the recombination coefficient at temperatures above, e.g., 700 K.

The rather limited range of temperatures of the cobalt catalyst is the result of practical limitations—the upper limit, at around 850 K, was the highest achievable temperature of the cobalt catalyst in our experimental setup. On the other hand, the lower limit was room temperature as our experimental system does not allow us to use any advanced cooling method on the cobalt catalyst. As a result of this, we did not reach temperatures where the cobalt oxide layer becomes unstable [20,21], thus achieving reliable and repeatable results. Furthermore, the industrial use of cobalt as a catalyst for different reactions usually occurs at elevated temperatures [40,41], which are encompassed in this study.

Figure 7 clearly shows that the recombination coefficient does not depend much on the pressure at low catalyst temperatures, up to approximately 500 K, but at higher temperatures, the recombination coefficient decreases with increasing pressure. This observation may explain the fact that Dickens et al., reported a very low recombination coefficient because they worked at a pressure as large as 13,333 Pa. In contrast, Cvelbar et al. [27] reported a coefficient about 30 times larger than Dickens et al., at a pressure of approximately 40 Pa. At higher pressures, Cvelbar et al., reported a decreasing recombination coefficient and stabilization at a value of approximately 0.085. Unfortunately, Cvelbar et al., did not report on the disk temperature upon measuring the recombination coefficients. The results shown in Figure 7 clearly show that the coefficient at an elevated pressure of approximately 200 Pa may assume any value between 0.05 and 0.15. The exact values depend significantly on the temperature of the catalytic disk.

The pressure range was chosen due to a few constraining factors. The first was the mass flow controller, which had a maximum flow rate of 3000 sccm. This resulted in a pressure in the measuring chamber of 214 Pa. While higher pressures could be achieved by limiting the pumping speed of the pump, the resulting pressures were highly inconsistent and were thus exempt from this study. At pressures lower than 42 Pa, the range of temperatures achievable by the cobalt probe was severely limited, and thus we limited ourselves to the specified pressure range. While the pressures chosen are not typical in industrial plasma applications [42,43], they still provide some useful data on the behavior of oxidized cobalt.

**Figure 8:**
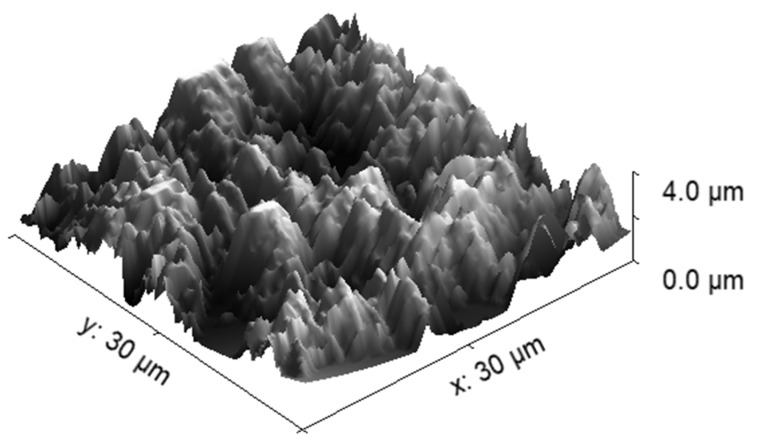
AFM image of a $30\times 30\;\mu m^{2}$ surface area of the oxidized cobalt disk. The RMS surface roughness was estimated to be $S_{q}=895\;nm$.

As mentioned earlier, the recombination coefficient is likely to depend on the surface morphology and composition. We processed our material at extreme conditions (at 1300 K in oxygen plasma) to obtain a stable film of cobalt oxide. The morphology as examined by atomic force microscopy (AFM) is shown in Figure 8. The AFM imaging was performed for the as-oxidized sample and oxidized sample treated with O atoms at several pressures and temperatures. No deviation from the as-oxidized sample was observed, thus confirming the stability of the oxide film throughout the experiments with O atoms. In Figure 8, one can observe a very rough surface with cobalt oxide structures of a vertical dimension exceeding 1 µm. The actual area of the well-oxidized cobalt surface is thus several times larger than the geometrical area. The roughness of the sample probed for the recombination coefficient probably explains the discrepancies in the absolute values as compared with some other reports [23,24,25,26,27]. As mentioned in the introduction, earlier authors reported various values ranging between 0.0025 and 0.25. Our measurements fall into the range between approximately 0.05 and 0.30 (Figure 5, Figure 6 and Figure 7). The lack of low values is explained by the high roughness of our samples because the recombination coefficient depends on the real, not geometric surface area.

The systematic measurements reported in this paper enable an insight into the mechanisms involved in the association of neutral oxygen atoms in the ground state on the surface of oxidized cobalt samples. Somewhat larger values than those reported by other authors are explained by rather large surface roughness, which develops upon oxidation at elevated temperatures. We performed the oxidation at the temperature of 1300 K for about 10 min to assure an approximately stable oxide film. All measurements of the recombination coefficient reported in this paper were performed in a temperature range between 300 and 850 K, which is well below the temperature used for the formation of the stable oxide film. The stability of the oxide film is crucial for the appropriate determination of the recombination coefficient because a rich morphology will develop on smooth metallic foil upon gradual oxidation at elevated temperatures.

It is well known that the surface association of oxygen atoms on solid materials follows two distinguished mechanisms, explained by the Eley–Rideal (ER) and Langmuir–Hinshelwood (LH) models. The ER model predicts an association of oxygen atoms impinging the surface with already adsorbed O atoms [44]. The association is instant, so there is no time for the accommodation of the impinging atoms on the surface. On the other hand, the LH model predicts the accommodation of atoms in the potential well of chemisorption on the surface, and the association of two adsorbed atoms [45]. The molecule formed on the surface, according to the LH model, then leaves the surface. The LH model requires the proximity of two adatoms to enable the formation of an oxygen molecule. The proximity is assured by oscillating the chemisorbed O atoms in the potential well of chemisorption. Because the amplitude of surface oscillations increases with increasing temperature, the recombination coefficient, according to the LH model, also increases with increasing temperature. Figure 6 reveals increasing γ with increasing temperature in the range from 300 to approximately 700 K, but the coefficient stabilizes at high temperatures. This stabilization can be explained by the saturation of the coefficient according to the LH model, i.e., almost all O atoms trapped in the chemisorption well are recombined at high temperatures. The concentration of adsorption sites on the solid material is limited, so the coefficient is well below 1 even for the surface with a very rich morphology (Figure 8). The coefficient should be lower for smooth surfaces, but well-oxidized metals are usually rough, so the values for polished samples would be misleading in practical cases.

**Figure 9:**
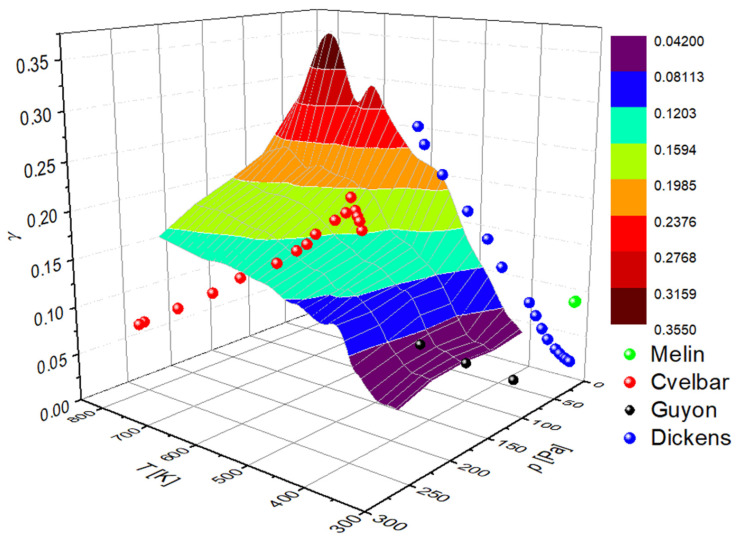
The recombination coefficient (γ) of the oxidized cobalt disk versus the temperature (T) of the cobalt disk and the pressure (p) inside the system.

A graphical presentation of the recombination coefficient is shown in Figure 9. The measured points reported by previous authors who mentioned the gas pressure and the sample temperature in their reports are also shown in Figure 9. There are not many of these, because most authors either failed to report both parameters or because they chose the pressures well above the range probed in this work. We performed systematic measurements in the range of 40–220 Pa because the O-atom density in reactors for the treatment of solid materials peaks in this range as long as the treatment is performed at highly non-equilibrium conditions in which the gas is close to room temperature and free from charged particles [46]. Additionally, in Table 1, the measured recombination coefficient of cobalt for oxygen atoms is compared with results from the literature.

Finally, it should be stressed that we chose the range of experimental conditions (in particular, pressure) in order to perform reliable measurements and interpret them spotlessly. Namely, in the range of pressures up to about 2 mbar, the gas-phase association of O atoms to oxygen molecules is negligible because such a reaction requires a three-body collision in order to satisfy the conservation of energy and momentum. Any attempt to perform measurements at an elevated pressure would require taking into account the gas-phase reactions, which would complicate the mathematical formalism, in particular Equation (3).

The systems employing cobalt oxide catalysis often operate at atmospheric pressure and above to take advantage of the large gas throughput. We limited our study to the heterogeneous surface association of O atoms to oxygen molecules. Practically important catalysts include other gases, and perhaps the most studied is the relatively low-temperature oxidation of carbon monoxide [47,48,49]. Numerous authors have reported high oxidation rates, but the coefficients in terms of the probability for an impinging radical (e.g., a CO molecule) to interact on the surface have rarely been reported since the atmospheric pressure gas dynamics include the concentration gradients, diffusion, and adsorption on available surface sites. The modeling of the gas kinetics at atmospheric pressure is complex as compared with the low-pressure systems. The results provided in this article thus illustrate the surface recombination efficiency in the limitation of low-pressure systems, such as diffusion-governed motion. Any extrapolation of the measured values is not scientifically justified but could be useful for the estimation of the reaction probabilities at elevated pressure. Figure 7 indicates a rather stable coefficient in the range of pressures between 100 and 200 Pa and temperatures between 450 and 550 K. The large deviation for the values obtained in this range of experimental conditions was observed at lower pressures and, especially, higher temperatures, which are not relevant for surface reactions such as the heterogeneous oxidation of carbon monoxide.

## 4. Conclusions

The paradox of large discrepancies between the measured values of the coefficient for the heterogeneous recombination of oxygen atoms on cobalt surfaces has been resolved. The coefficient depends on the roughness and thus the real surface area, as well as the temperature of the oxidized cobalt foil and the pressure in the experimental chamber. We oxidized the foil during exposure to oxygen plasma at a temperature of 1300 K for about 10 min in order to assure a stable oxide film during the measurements of the recombination coefficient. The recombination coefficient increases monotonously with increasing surface temperature, which has been explained by increasing the mobility of adsorbed O atoms and thus increasing the probability for surface association due to the Langmuir–Hinshelwood model. The recombination coefficient somehow decreases with increasing pressure in the range of pressures between 40 and 200 Pa, which might be explained by the screening effect of physisorbed O_2_ molecules and thus suppressing the direct association of O atoms impinging the surface covered by the chemisorbed O atoms, i.e., the Eley–Rideal model. Knowledge of the variation in the recombination coefficient is necessary to understand the surface kinetics involved in atomic layer oxidation of both inorganic and organic materials.

## Figures and Tables

**Table 1:** Comparison of measurements with various literature for the recombination coefficient (γ) of a cobalt surface for neutral oxygen atoms at various pressure (p) and temperature (T) ranges.

$p\left[Pa\right]$	$T\left[K\right]$	γ	Reference
42–214	370–850	0.04–0.35	this article
650	300	0.00049	[23]
4	300–625	0.0018–0.25	[24]
1–4	300	0.075	[25]
110	300–473	0.029–0.034	[26]
10–400	750	0.08–0.14	[27]

## Data Availability

The data that support the findings of this study are available upon reasonable request from the authors.
